# Species-Level Spatio-Temporal Dynamics of Cyanobacteria in a Hard-Water Temperate Lake in the Southern Baltics

**DOI:** 10.3389/fmicb.2021.761259

**Published:** 2021-10-29

**Authors:** Ebuka Canisius Nwosu, Patricia Roeser, Sizhong Yang, Sylvia Pinkerneil, Lars Ganzert, Elke Dittmann, Achim Brauer, Dirk Wagner, Susanne Liebner

**Affiliations:** ^1^Section Geomicrobiology, GFZ German Research Centre for Geosciences, Potsdam, Germany; ^2^Marine Geology Section, Leibniz Institute for Baltic Sea Research (IOW), Rostock, Germany; ^3^Section Climate Dynamics and Landscape Evolution, GFZ German Research Centre for Geosciences, Potsdam, Germany; ^4^Institute of Biochemistry and Biology, University of Potsdam, Potsdam, Germany; ^5^Institute of Geosciences, University of Potsdam, Potsdam, Germany

**Keywords:** *Cyanobium*, picocyanobacteria diversity, amplicon sequencing, lake monitoring, ecological succession, lake stratification, psychrotolerant

## Abstract

Cyanobacteria are important primary producers in temperate freshwater ecosystems. However, studies on the seasonal and spatial distribution of cyanobacteria in deep lakes based on high-throughput DNA sequencing are still rare. In this study, we combined monthly water sampling and monitoring in 2019, amplicon sequence variants analysis (ASVs; a proxy for different species) and quantitative PCR targeting overall cyanobacteria abundance to describe the seasonal and spatial dynamics of cyanobacteria in the deep hard-water oligo-mesotrophic Lake Tiefer See, NE Germany. We observed significant seasonal variation in the cyanobacterial community composition (*p* < 0.05) in the epi- and metalimnion layers, but not in the hypolimnion. In winter—when the water column is mixed—picocyanobacteria (*Synechococcus* and *Cyanobium*) were dominant. With the onset of stratification in late spring, we observed potential niche specialization and coexistence among the cyanobacteria taxa driven mainly by light and nutrient dynamics. Specifically, ASVs assigned to picocyanobacteria and the genus *Planktothrix* were the main contributors to the formation of deep chlorophyll maxima along a light gradient. While *Synechococcus* and different *Cyanobium* ASVs were abundant in the epilimnion up to the base of the euphotic zone from spring to fall, *Planktothrix* mainly occurred in the metalimnetic layer below the euphotic zone where also overall cyanobacteria abundance was highest in summer. Our data revealed two potentially psychrotolerant (cold-adapted) *Cyanobium* species that appear to cope well under conditions of lower hypolimnetic water temperature and light as well as increasing sediment-released phosphate in the deeper waters in summer. The potential cold-adapted *Cyanobium* species were also dominant throughout the water column in fall and winter. Furthermore, *Snowella* and *Microcystis*-related ASVs were abundant in the water column during the onset of fall turnover. Altogether, these findings suggest previously unascertained and considerable spatiotemporal changes in the community of cyanobacteria on the species level especially within the genus *Cyanobium* in deep hard-water temperate lakes.

## Introduction

Photosynthetic microorganisms function as the basis of food chains in aquatic environments and are thus of importance to the ecosystem and to humans. Cyanobacteria have been studied extensively as an important contributor to global primary production in oceans and lakes (Mazard et al., 2016; Soule and Garcia-Pichel, 2019). In temperate climate, most lakes are stratified usually beginning in late spring into the epilimnion (warmer upper water column), the hypolimnion (dark and cold deep water), and the metalimnion, a transition layer between the warmer and colder layers. Because of the fast changes in temperature and increasing density within the metalimnion, this layer acts as a physical barrier between the epi- and hypolimnion (Boehrer and Schultze, 2008). One of the expected effects of increasing global temperatures and climate change on temperate lakes is that prolonged periods of stratification may extend and thus change the community structure of cyanobacteria (diversity and composition) inhabiting these lakes (Salmaso et al., 2015; Visser et al., 2016; Huisman et al., 2018).

Molecular-based techniques have been employed in studying seasonal changes in lacustrine bacterial communities (e.g., Allgaier and Grossart, 2006; Ivanikova et al., 2007) and the interactions between bacteria and phytoplankton in lakes (Nitin Parulekar et al., 2017; Schweitzer-Natan et al., 2019). Studies on lake bacterioplankton seasonality based on 16S rRNA gene sequencing have for instance brought attention to the sources and dangers of eutrophication via nutrient loading (e.g., Kong et al., 2019). Furthermore, advances in sequencing techniques with improved taxonomic resolution have provided additional understanding to the seasonal succession of bacterioplankton communities in the upper waters of lakes (Okazaki and Nakano, 2016; Diao et al., 2017; Salmaso et al., 2018). For example, in Lake Vechten (Netherlands; max. depth 11 m), high-throughput sequencing of every meter in the water column revealed changes in bacteria community composition to be driven by lake oxygenation and sulphidic conditions (Diao et al., 2017). Other factors driving cyanobacteria seasonality and niche differentiation include light intensity and differences in cyanobacteria accessory pigments which influence their adaptation to spectral light quality (Pick and Agbeti, 1991; Vörös et al., 1998; Callieri et al., 2012b). Research on seasonality and succession of phytoplankton in lakes either via cell counts or the creation of 16S rRNA sequence clone libraries approaches have helped in the identification of important taxonomic groups within the phylum Cyanobacteria (Albrecht et al., 2017; Butts and Carrick, 2017). An advantage of using high-throughput sequencing techniques is the possibility of a more holistic estimation, especially of small (0.2–2 μm) picocyanobacteria (e.g., *Synechococcus* sp. and *Cyanobium* sp.). Members of this group are often not reliably identified and differentiated by microscopy (Callieri et al., 2012b; Salmaso et al., 2018). Except for Salmaso et al. (2018) that sequenced bacterial 16S rRNA gene to analyze annual bacterioplankton community composition in the euphotic zone (up to 21 m water depth) of Lake Garda (max. water depth 350 m), there are, to the best of our knowledge, no spatiotemporal comparative studies of cyanobacterial succession across different water layers and seasons in deep temperate lakes and in combination with cyanobacteria-specific marker gene analysis.

In this study, we integrate highly resolved sampling throughout the year 2019 with high-throughput amplicon sequencing and quantitative PCR to unravel seasonal succession dynamics of pelagic cyanobacteria community composition (CCC) and abundance as well as possible niche segregation in Lake Tiefer See near Klocksin (TSK). We aim to understand the spatiotemporal succession dynamics of cyanobacteria and their potential lake environmental drivers in deep, temperate hard-water lakes. Lake Tiefer See is ideal for this study because with 62 m water depth it belongs to the deepest lakes in northeastern Germany. In addition, since the beginning of the last decade, Lake Tiefer See has been the focus of an extensive and high-resolution climate monitoring program as well as several paleolimnological climate reconstruction investigations (e.g., Dräger et al., 2019; Nwosu et al., 2021a; Roeser et al., 2021).

## Materials and Methods

### Study Site

**FIGURE 1:**
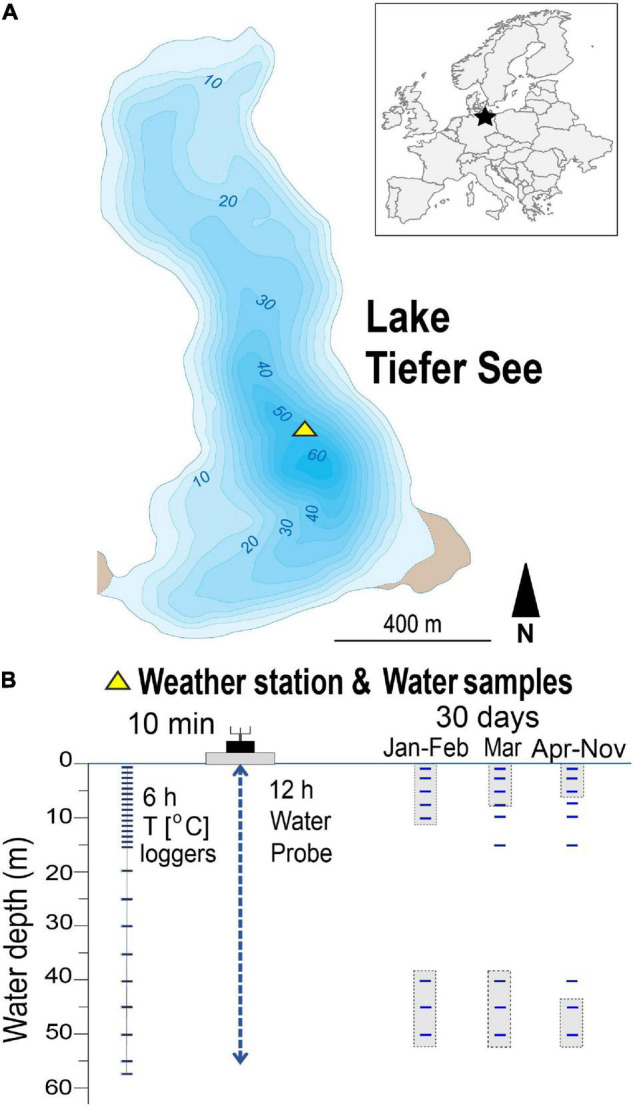
Location of the study site and sampling setup. **(A)** Location of Lake Tiefer See near Klocksin (TSK, NE Germany). **(B)** Scheme of lake monitoring setup showing depths of water column temperature loggers and the depths from which water samples were taken. Shaded sampling points indicate samples that were pooled for genomic DNA extraction.

The hard-water Lake Tiefer See is located in the natural park “Nossentiner/Schwinzer Heide” in the southern Baltic lowlands (Figure 1A). The lake basin was formed during the last glaciation as part of a subglacial channel system in a morainic terrain and the present lake is part of the Klocksin-Lake-Chain (Dräger et al., 2017). The lake has a maximum depth of 62 m and no major inflow and outflow with a surface area of about 0.75 km^2^, and catchment area of about 5.5 km^2^ dominated by glacial till. The area around Lake Tiefer See is characterized by a warm-temperate climate at the transition from oceanic to continental conditions. Mean monthly air temperatures range from 0°C in January to 17–18°C in July with maxima up to 30°C and minima down to −5°C. Mean monthly precipitation varies between ∼ 40 mm during winter and ∼ 60 mm in summer with a mean annual precipitation of 560–570 mm (Roeser et al., 2021). Although the catchment is mainly used for agriculture (Theuerkauf et al., 2015), the direct shore-line of the lake is covered by a fringe of trees and there is no anthropogenic infrastructure such as buildings and roads at the lakeshore. The present-day lake status is oligo-mesotrophic and the circulation mode is either mono- or dimictic, depending on the formation of a winter ice cover (Dräger et al., 2017).

### Collection of Water Samples

Water samples were taken monthly between 30th January and 28th November 2019 close to the spot of the lake’s maximum depth (∼62 m; Figure 1A), where the floating weather monitoring station is also installed. The sampled water depths were 1, 3, 5, 7, 10, 15, 20, 40, 45, and 50 m. A sample volume of 250 mL lake water was collected for the upper water depths of 1, 3, 5 m and pooled (750 mL), representing the epilimnion. Likewise, volumes of 375 mL were collected for the lower water depths 45 and 50 m and pooled (750 mL), representing the hypolimnion. For each of the other depths 7, 10, 15, 20, and 40 m, respectively, 750 mL water samples were collected. The samples were collected in sterile glass bottles (Schott Duran <^®^, Germany), which were first flushed with lake water from the respective depths before samples were collected and transported in cold dark boxes to the laboratory where they are stored in refrigerators. They were then, along with blank water (autoclaved deionized water), filtered within 24 h after fieldwork using 0.2 μm cellulose filters (Sartorius AG, Germany) and stored at −20°C until nucleic acid extraction (Kurmayer et al., 2017).

### Lake Physicochemical Parameters

Measurements of the physicochemical parameters pH, dissolved oxygen (DO), turbidity, conductivity, and chlorophyll-a (Chl*a*) in the water column were conducted automatically using a multi-parameter water quality probe (YSI 6600 V2, Yellow Springs United States) in 0.5–1-m steps and 12-h resolution. The Chl*a* is used here as an indicator of phytoplankton biomass production. Owing to technical problems, data in February were only collected on 2 days (1st and 28th), and in March no data were recorded between the 2nd and the 10th. Water temperature was measured using 26 depth-stationary data loggers (HOBO Water Temp Pro v2, Onset United States) in 1-m steps from 0 to 15 m and 5-m steps from 15 to 55 m water depth (Figure 1B).

Water samples for molecular analyses, nitrate (NO_3_^–^) and total dissolved phosphorus (TDP) analyses were collected monthly. Nitrate measurements were done at the German Research Centre for Geosciences (GFZ) while TDP was determined at the Leibniz Institute for Baltic Sea Research Warnemünde (IOW). Nitrate was measured by suppressed ion chromatography using a SeQuant SAMS anion IC suppressor (EMD Millipore, Billerica, Massachusetts), a S5200 sample injector, a 3.0 × 250 mm LCA 14 column and a S3115 conductivity detector (all Sykam, Fürstenfeldbruck, Germany). The eluent was 5 mM Na_2_CO_3_ with 20 mg L^–1^ 4–hydroxybenzonitrile and 0.2% methanol. The flow rate was set to 1 mL min^–1^ and the column oven temperature to 50°C. Detection and quantification limits were calculated based on signal-to-noise (S/N) ratios of 3 and 10, respectively. All samples were measured in triplicates and every 10 injections a standard was measured to check for drift. Reproducibility was always better than 5% and the detection limit ranged between 1 and 4 μM.

For TDP, the water samples were immediately filtered using 0.45 μm syringe filters and acidified with sub-boiled HNO_3_ to 2%Vol. The TDP was measured by inductively coupled plasma optical emission spectrometry (ICP-OES; iCAP 7400, Duo, Thermo Fisher Scientific) using external calibration and Sc as internal standard. Precision and trueness were checked with the international reference material SLRS-6 (NRCC) spiked with 20 μg L^–1^ P and were 9.6 and 5.7%, respectively.

### Extraction of DNA

Nucleic acid extractions were carried out in a clean laboratory where no polymerase chain reaction (PCR) had been performed prior to the extraction, following established methods and precautions to limit contamination (Nwosu et al., 2021b). Because of the mixed water column in January and February, water samples for molecular analyses from the water depths 1, 3, 5, 7, 10 m were pooled, and reported as the mean depth (5 m; Figure 1B). The water depths 40, 45, 50 m were equally pooled, and reported as the mean depth (45 m). In March, when temperatures began to increase, four depths in the water column were reported as follows: 5 (1, 3, 5, and 7 m pooled), 10, 20, and 45 m (40, 45, and 50 m pooled). Genomic DNA (gDNA) of water samples, blank water filtrations and blank DNA extraction using autoclaved deionized water were extracted from filters using the DNeasy PowerWater Kit (QIAGEN, United States) following the manufacturer’s specifications. The extracted gDNA was eluted in 100 μL elution buffer, quantified with the Qubit dsDNA HS Assay Kit (Invitrogn, United States) and stored at −20°C pending downstream analysis.

### Quantification of Cyanobacteria Abundance

Total cyanobacteria were quantified with a SYBRGreen quantitative PCR (qPCR) assay amplifying specifically the cyanobacteria 16S rRNA-ITS (internal transcribed spacer) region ca. 350 bp using the primers CSIF (5′-GYCACGCCC GAAGTCRTTAC-3′) and 373R (5′-CTAACCACCTGAGCT AAT-3′) (Janse et al., 2003). The final volume of the qPCR reactions was 20 μL containing 10 μL of KAPA SYBR <^®^ FAST qPCR Master Mix (Sigma-Aldrich, Germany), 0.2 μL of each forward and reverse primer (100 μM, Biomers), 5.8 μL PCR water and 4 μL of template DNA. The qPCR program consisted of an initial polymerase activation step (95°C for 15 min), followed by 40 cycles of denaturation at 94°C for 60 s, annealing at 60°C for 60 s and extension at 72°C for 60 s. The qPCR program was followed by a melting curve step from 70 to 95°C at a transition rate of 1°C per 5 s to determine the specificity of the amplification. The amplified products were confirmed with agarose gel electrophoresis. Tenfold dilution standards were prepared from gDNA of *Synechocystis* for each qPCR assay, ranging from 4.7 × 10^7^ to 4.7 × 10^3^ copies ng^–1^ DNA to create the standard curves from which the quantification cycle (*Cq*) values were determined (Bustin et al., 2009). All qPCR assays were performed in triplicates on a CFX96 real-time thermal cycler (Bio-Rad Laboratories Inc., United States). The copy numbers of the 16S rRNA-ITS region were calculated after Savichtcheva et al. (2011). The obtained values were the mean triplicates of each sample expressed as cyanobacterial abundance normalized to extracted DNA (copies ng^–1^ DNA) with a minimum and maximum quantification efficiency of 91 and 94%, respectively.

### Sequence Libraries Preparation

Cyanobacteria-specific primers CYA359F (5′-CGGACGGGTG AGTAACGCGTG-3′) and CYA784R (5′-ACTACWGGGGTA TCTAATCCC-3′) (Nübel et al., 1997) that amplify a > 400-nt-long fragment of the V3 - V4 regions of the 16S rRNA gene were used for preparing two Illumina high-throughput sequencing (HTS) libraries. The primers had unique tags that served to differentiate the samples. The samples and negative controls (that is, a reaction with PCR water as template) were amplified in a 50 μL volume PCR reaction, comprising 10X Pol Buffer C (Roboklon GmbH, Berlin, Germany), 25 mM MgCl_2_, 0.2 mM deoxynucleoside triphosphate (dNTP) mix (Thermo Fisher Scientific), 0.5 mM of each primer (TIB Molbiol, Berlin, Germany), and 1.25 U of Optitaq Polymerase (Roboklon). The volume of the template DNA used in each reaction varied between 1 and 4 μL depending on the genomic DNA concentration. The PCR program included a first denaturation step at 95°C for 10 min, followed by 35 cycles at 95°C for 15 s, annealing at 60°C for 30 s, extension at 72°C for 45 s and a final extension step at 72°C for 5 min. To avoid cross-contamination, the PCR reactions were done monthly, after each sampling. Furthermore, to control for reproducibility of the PCR and sequencing results, all samples were amplified in a second PCR run (technical replicates). The tagged PCR products were then purified with the Agencourt AMPure XP kit (Beckman Coulter, Switzerland) and eluted in 30 μL DNA/RNA-free water. The purified product was quantified with a Qubit 2.0 Fluorometer. Equimolar concentrations of all samples and negative purified PCR controls were pooled into a multiplex library (*n* = 48 samples and 2 negative controls). Similarly, equimolar concentrations of all sample technical replicates including negative controls were pooled into a second multiplex library (*n* = 48 technical replicates and 2 negative controls). The libraries were paired-end sequenced (2 × 300 bp) on an Illumina MiSeq system at Eurofins Scientific (Constance, Germany).

### Bioinformatics

The obtained 10,546,152 sequence reads were quality checked on a raw fastq file with FastQC (Andrews et al., 2015). The reads were demultiplexed, first by using the “make.contigs” function in Mothur (v.1.39.5: pdiff = 2, bdiff = 1, and default setting for others) (Schloss et al., 2009). Based on the report files generated by “make.contigs” function, the sequence identifiers were retrieved for those sequences with minimum overlap (length > 25), maximum mismatches (<5), and the maximum number of ambiguous bases of zero (which means there is no base marked with “N”). According to the sequence identifiers from the previous step the sequences were extracted with “filterbyname.sh” function from BBtools (Bushnell et al., 2017) from the raw paired-end fastq file. This step was followed by correcting the sequence orientation and removing sample unique barcodes by using extract_barcodes.py in QIIME1 (Caporaso et al., 2010) and finally, the primers were removed using awk script (Aho et al., 1979). The demultiplexed sequences were fed to DADA2 (Callahan et al., 2016) for filtering, dereplication, chimera check, sequence merge, and ASV (amplicon sequence variants) calling. The output of DADA2 was further fed to QIIME2 (Bolyen et al., 2019) for taxonomic assignment against the SILVA138 database (Quast et al., 2013).

### Data Availability

Sequencing data and metadata are deposited at the European Nucleotide Archive (ENA) under Bio Project accession number PRJEB40406 as well as sample accession numbers ERS5083566–ERS5083644.

### Sequence Data Processing and Statistics

The two libraries were merged together by taking the average of their relative abundances. Bubble plots were used to illustrate differences in seasonal and spatial cyanobacteria assemblage and produced with the free software tool^1^. Alpha and beta diversity estimations, Spearman correlation and multivariate permutational analysis of variance (PerMANOVA) were performed using the PAST v4.01 software (Hammer et al., 2001). Two-way analysis of variance (ANOVA) tests, non-metric multidimensional scaling (NMDS) and distance-based redundancy analysis (dbRDA) were performed using the “vegan” package in R (Oksanen et al., 2019). Prior to alpha diversity calculation (richness, Shannon diversity and Pielou’s evenness) the ASV read counts were rarefied to account for differences in sequencing depth (2,200 read counts per sample) using the “rtk” package in R (Saary et al., 2017). Also, before beta diversity measurements, the ASV cut-off was set to 0.1% to eliminate very rare taxa. The statistical significance level for uni- and multivariate statistical analyses was set to < 0.05. To determine which lake factors have a significant impact on cyanobacteria spatiotemporal alpha diversity dynamics a two-way ANOVA (additive and interactive models) test was used to evaluate the effect of seasons and stratification zones (epi-, meta- and hypolimnion) on cyanobacteria species richness and evenness. In the ANOVA models, species richness or evenness was the response variable while season and stratification zones were the predictors. The seasons were defined based on changes in the water column as reveled by multi-parameter probe data. The ANOVA models’ assumptions were evaluated with Tukey’s-honestly-significant-difference (TukeyHSD) *post hoc* tests. An NMDS ordination based on the Bray-Curtis dissimilarity matrix was used to determine whether the samples at the ASV level showed distinct grouping patterns. To assess whether the grouping patterns of cyanobacterial communities at the ASV level as revealed by the NMDS were significantly different from each other a non-parametric PerMANOVA test based on Bray-Curtis matrix using seasons as predictors was conducted. Hellinger-transformed cyanobacteria absolute read counts data were used in the NMDS analysis and PerMANOVA test (Legendre and Gallagher, 2001). To align pooled water samples with depth-specific environmental variables, the averages of the environmental variables from the corresponding water depths were calculated. For example, for the reported mean water depth 5 m (water depths 1, 3, 5, 7, and 10 m) in January, average water temperature from the depths 1, 3, 5, 7, 10 m on the day of sample collection was aligned to the reported mean sample depth 5 m. To assess the correlation of *Cyanobium* ASVs 0005, 0008, and 0014 abundance to environmental parameters a Spearman’s rank correlation coefficient was calculated. Prior to the correlation analysis the environmental data (predictors) were standardized by subtracting the mean and dividing by the standard deviation (Z-Score) and the *Cyanobium* ASVs 0005, 0008, and 0014 (response variables) read counts were Hellinger transformed. Standardized environmental data were also fitted into the dbRDA model. In this model, environmental and cyanobacteria community data were the explanatory and response variables, respectively. The dbRDA analysis was performed using the “capscale” function and Bray-Curtis distance in “vegan”. The model and axes significance were tested by a Monte Carlo permutation test (*n* = 999) using the “anova.cca” function in “vegan”. Collinearity in the explanatory variables was tested with a variance inflation factor (VIF) using the “vif.cca” function in “vegan”. Explanatory variables were then additively tested until only those with a VIF score < 10 remained. The significant subset of explanatory variables that may explain the variability of cyanobacterial community composition was determined via forward selection using the function “ordiR2step” function in “vegan”. Variation partitioning analysis (VPA) (Legendre et al., 2005) with redundancy analysis (RDA) was implemented on two categories of explanatory variables, that is, physicochemical parameters (temperature, pH, turbidity, conductivity, and DO) and nutrients (TDP and NO_3_^–^) to identify their unique and interactive effects on the cyanobacteria population dynamics (response variable). The significance of the fractions of variability explained by the categories was tested and determined via a Monte Carlo permutation test (*n* = 999) in CANOCO5 (Smilauer and Leps, 2014).

## Results

### Water Column Physicochemical Parameters

**FIGURE 2:**
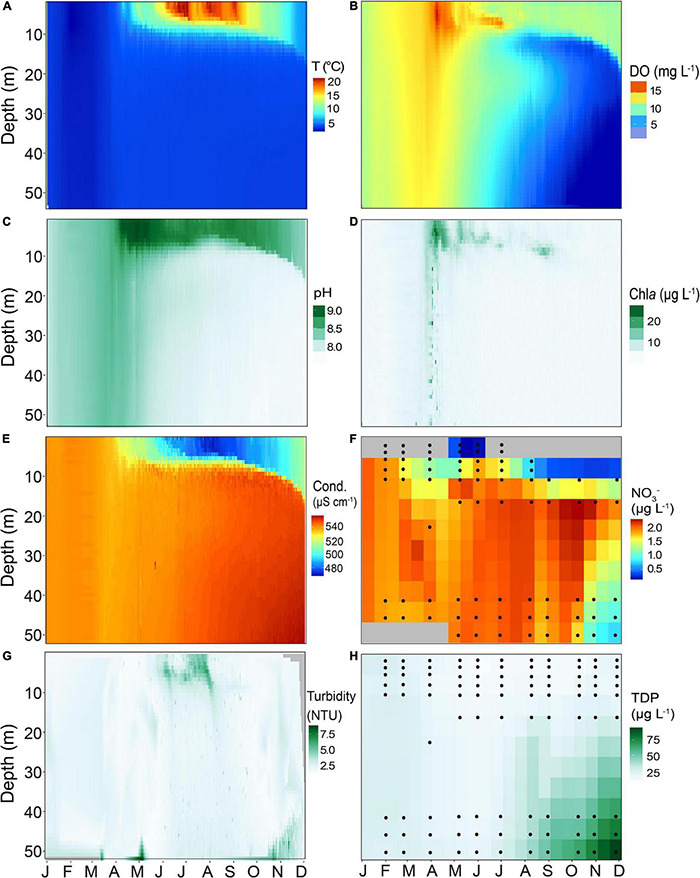
Heatmaps of different physicochemical parameters of Lake Tiefer See for the year 2019 measured in the water column using a multi-parameter probe (YSI 6600 V2), showing **(A)** Temperature (T), **(B)** Dissolved oxygen (DO), **(C)** pH, **(D)** Chlorophyll a (Chl*a*), **(E)** Conductivity (Cond.), **(F)** Nitrate (NO_3_^–^), **(G)** Turbidity, and **(H)** Total dissolved phosphorus (TDP). Probe data were interpolated for 1 day and 1 m water depth. Black circles in NO_3_^–^ and TDP heatmaps represent the depths from which measurements were taken. The latter were interpolated to 15 days and 5 meters water depth. Gray fields in NO_3_^–^ heatmap are depths with no data.

Temperature and dissolved oxygen (DO) in the water column of Lake Tiefer See (Figures 2A,B) showed that in 2019 thermal stratification began in early April and continued beyond late November. Highest epilimnion temperatures (>22°C) were recorded during summer months. In the epilimnion (0–6 m) three periods with high temperatures followed by cooling of ∼5°C drop occurred. The first lasted the entire month of June (*T*_*max*_ = 21.6°C, *T*_*average*_ = 20°C) while the second phase covered the last week of July until the first week of August (*T*_*max*_ = 20.9°C, *T*_*average*_ = 19°C). The last warm period was recorded from late August to the first few days in September (*T*_*max*_ = 20.7°C, *T*_*average*_ = 17°C). Oxygen depletion in the bottom water started circa 3 weeks after the onset of thermal stratification and the onset of pelagic productivity. The DO reached minimum values (∼0.67 mg L^–1^) at 40–50 m water depth between October and November (Figure 2B). Additionally, Lake Tiefer See developed a zone of metalimnetic oxygen minimum between 10 and 13 m from June to September. Enhanced pH and Chl*a* values (Figures 2C,D) reflect the first algal-blooms in mid-April between 1 and 7 m water depth, shortly before the onset of lake stratification. From late March until mid-July, peak Chl*a* were observed at approximately 7 m water depth, and at 10 m water depth from August to September (Figure 2D). In winter to early spring, conductivity in the mixed water column ranged between 530 and 540 μS cm^–1^ (Figure 2E). During lake stratification, conductivity was lowest in the epilimnion (<480 μS cm^–1^; 1–7 m) and highest in the meta- and hypolimnion (>540 μS cm^–1^; 10–60 m). Nitrate concentrations varied between 1 and 2 μg L^–1^, except in October and November where they reached only 0.2 μg L^–1^ (Figure 2F). Turbidity peaked between 5 and 6 NTUs in summer in the upper water column (down to 10 m) likely as a combined result of primary productivity and calcite precipitation (Figure 2G). TDP values were generally higher in the hypolimnion, gradually increasing from July through November reaching up to ∼70 μg L^–1^ in the bottom waters (Figure 2H). In contrast, the TDP in the epilimnion ranged between 10 and 15 μg L^–1^, except for January and February, when the lake was in an isothermal state and the TDP values were between 20 and 25 μg L^–1^ throughout the mixed water column.

### Cyanobacterial Community Composition

**FIGURE 3:**
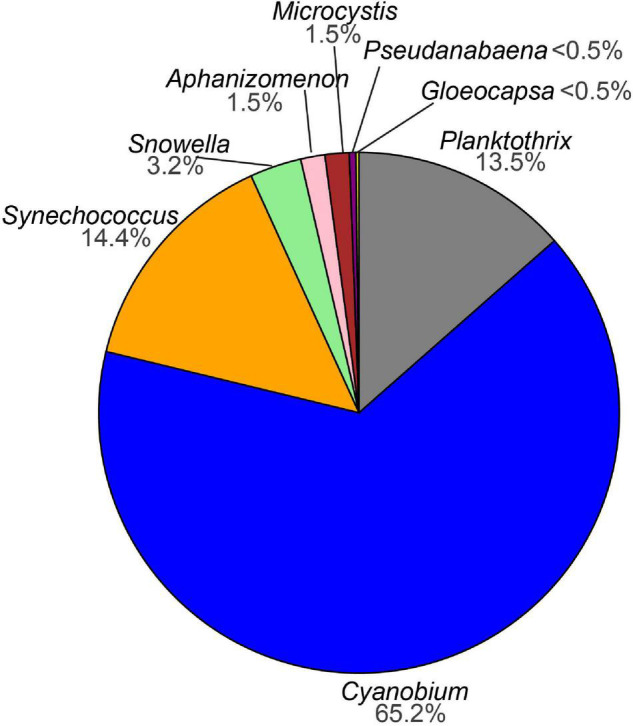
Pie chart showing the percentage of amplicon sequence variants (ASVs) assigned to cyanobacteria (>0.1% relative abundance) from this study.

Sequencing of the water samples resulted in a total of 4,864,912 denoised and error-corrected absolute read counts which DADA2 inferred into 1599 ASVs. The two negative extraction controls that were included in the sequencing run each comprised less than 1% of the reads (17 ASVs) compared with the sample average and were removed. After ASVs assigned to chloroplasts, non-photosynthetic cyanobacteria (the orders Sericytochromatia and Vampirivibrionia) and other bacteria had been removed, 894 ASVs with a total 2,915,251 read counts remained. Next, global singletons and doubletons (492 ASVs) as well as 201 ASVs that occurred in fewer than three samples over the whole dataset were removed to reduce influence from very rare taxa. The filtered dataset comprised 201 ASVs assigned to photosynthetic cyanobacteria with a combined 2,901,116 absolute read counts and distributed across 96 samples (Supplementary Table 1). Of the 201 ASVs, two were assigned to order level (Cyanobacteriales), six were assigned to family level (2x Leptolyngbyaceae, 2x Coleofasciculaceae, Microcystaceae, Cyanobiaceae), and 193 were assigned to genus level. The ASVs of the genera *Planktothrix, Cyanobium, Synechococcus, Snowella, Aphanizomenon, Microcystis, Pseudanabaena*, and *Gloeocapsa* were most abundant. Of the cyanobacterial ASVs with 0.1% relative abundance, 65% were assigned to the genus *Cyanobium* (Figure 3 and Supplementary Figure 1).

### Cyanobacterial Seasonal Variation

**FIGURE 4:**
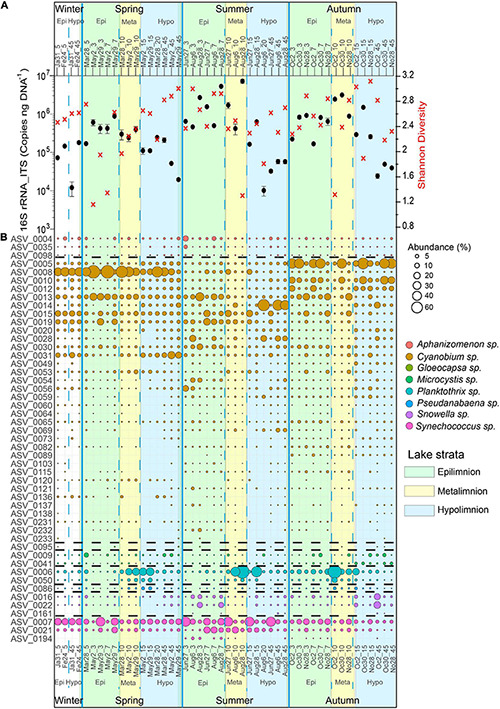
Cyanobacteria community structure, abundance and diversity in Lake Tiefer See throughout the year 2019. **(A)** Cyanobacterial abundance quantified via qPCR (black dots; Error bars, which are hidden by the dots in some cases, give the standard deviations for three independent amplifications.) and Shannon diversity (red crosses). **(B)** Bubble plot showing the spatiotemporal variation of cyanobacteria community composition at the amplicon sequence variant level (cutoff > 0.1% relative abundance). Numbers beside months refer to sampling date while number after the underscore refers to water depth, e.g., May2_10 refers to a sample collected at 10 m water depth on 2nd May. Epi = epilimnion, Meta = metalimnion, Hypo = hypolimnion. Taxa follow the same order as in the legend and broken lines indicates where the bubbles of one taxon end and the next begins.

A general trend in cyanobacterial 16S rRNA-ITS gene copy numbers was observed with highest mean cyanobacteria abundances in summer (1.2 × 10^6^ copies ng^–1^ DNA, *n* = 18) and fall (6.7 × 10^5^ copies ng^–1^ DNA, *n* = 18) throughout the water column. In contrast, lower mean cyanobacteria abundances were observed in spring (3.2 × 10^5^ copies ng^–1^ DNA, *n* = 16) and winter (9.4 × 10^4^ copies ng^–1^ DNA, *n* = 4), respectively, throughout the water column. The highest average numbers of cyanobacterial 16S rRNA-ITS gene copies were measured in the epi- and metalimnion in summer (2.2 × 10^6^ copies ng^–1^ DNA, 1–10 m, *n* = 9) and fall (1.1 × 10^6^ copies ng^–1^ DNA, 1–10 m, *n* = 9), while the hypolimnion had the lowest numbers throughout the different seasons, except for winter (Figure 4A).

The spatiotemporal variation was mainly driven by the ASVs belonging to the cyanobacterial orders Synechococcales (*Cyanobium, Snowella*, and *Synechococcus*), Nostocales (*Aphanizomenon*), Chroococcales (*Microcystis*), and Oscillatoriales (*Planktothrix*) (Figure 4B). The seasonal and spatial dynamics of the ASVs assigned to the most dominant taxa (>1% relative abundance) in Lake Tiefer See during the study period were as follows. In Winter (January - February), ASV0008 (*Cyanobium*), and ASV0007 (*Synechococcus*) were the most abundant (≥30%) cyanobacteria species throughout the water column. In Spring (March–May), *Cyanobium* ASV0008 bloomed in the epi- and metalimnion exceeding a relative abundance of 40%. At the same time, in the metalimnion the abundance of *Planktothrix* ASV0006 increased more than fivefold to ≥ 30% and was maintained high until late autumn. In the hypolimnion, ASV0004 (*Aphanizomenon*), and ASV0009 (*Microcystis*) increased only slightly (≥5%) in spring. In summer, *Aphanizomenon* ASV0004 (June) and the two *Snowella* ASVs 0016 and 0022 (August) behaved similarly with increase in abundance from ∼2 to 11% in the epilimnion. *Synechococcus* ASV0007 abundance increased to more than 30% throughout the water column in June. Starting in August, ASVs 0006 and 0014 assigned to *Planktothrix* and *Cyanobium*, respectively, subsequently became the most dominant ASVs (≥40%) in the meta- and hypolimnion, respectively. In fall (October), *Cyanobium* ASV0005 was the most abundant (≥40%) in the epi- and hypolimnion. In the metalimnion (10 m), *Planktothrix* ASV0006 abundance was higher (≥40%) than that of *Cyanobium* ASV005 (∼30%). Also, the *Microcystis* abundance increased throughout the water column from 2 to 6% whereas the *Snowella* ASVs 0016 and 0022 increased by more than 20% in the hypolimnion only.

The CCC at the ASV level showed distinct spatial and temporal clustering patterns as shown by NMDS (Supplementary Figure 2), and confirmed by a one-way PerMANOVA test to be significantly different from each other (*p* < 0.05; *F* = 10.72; Supplementary Table 2).

### Cyanobacterial Alpha Diversity Indices

A two-way ANOVA was used to determine if possible differences in cyanobacteria species richness were driven by seasonality, lake stratification (epi-, meta-, and hypolimnion) and/or an interaction effect of both factors. Species richness was found to be significantly impacted by season [*F*(3) = 8.046, *p* < 0.05; Figure 4], but not by depth nor an interaction between both terms. A Tukey *post hoc* test revealed significant pairwise differences in cyanobacteria species richness between spring and summer (+5.8 species richness; Supplementary Table 3), spring and autumn (−8.6 species richness), and between epi- and hypolimnion depths (−3.7 species richness). Cyanobacteria species evenness was not significantly impacted by the seasonal and lake stratification factors (Supplementary Figure 3).

**FIGURE 5:**
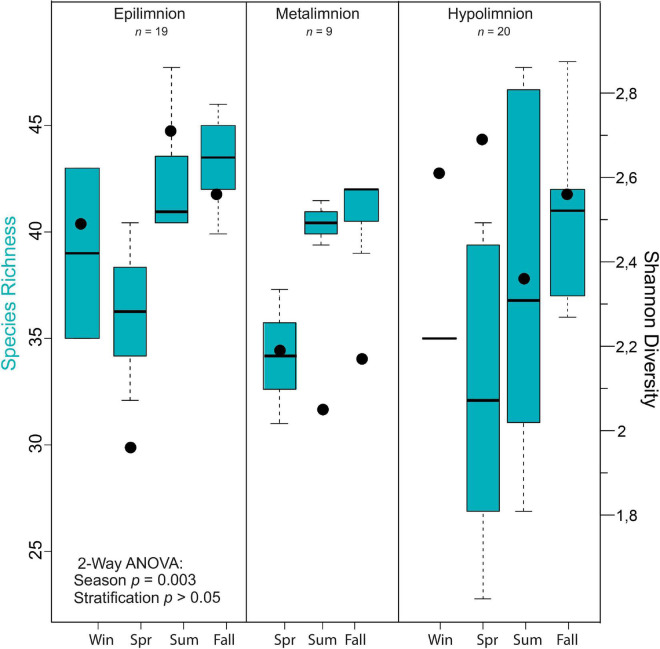
Alpha diversity of cyanobacteria communities in Lake Tiefer See. Box plots showing the results of seasonal and stratification effects on cyanobacteria richness in Lake Tiefer See via a two-way analysis of variance (ANOVA) analysis and Shannon diversity indices shown by black dots. The ANOVA models’ assumptions were evaluated with Tukey’s honestly-significant-difference (TukeyHSD) *post hoc* tests.

In general, mean cyanobacterial species diversity across the seasons as revealed by Shannon index was lowest in the metalimnion compared to the epi- and hypolimnion (Figures 5, 4A). In the epilimnion, diversity was highest in summer (2.7) and lowest in spring (2.0). Conversely, diversity decreased slightly from spring to summer from 2.2 to 2 in the metalimnion and 2.7 to 2.3 in the hypolimnion. Cyanobacteria diversity in the metalimnion from March to November was between 2.1 and 2.2.

### Relationship Between Certain *Cyanobium* Amplicon Sequence Variants and Environmental Variables

**FIGURE 6:**
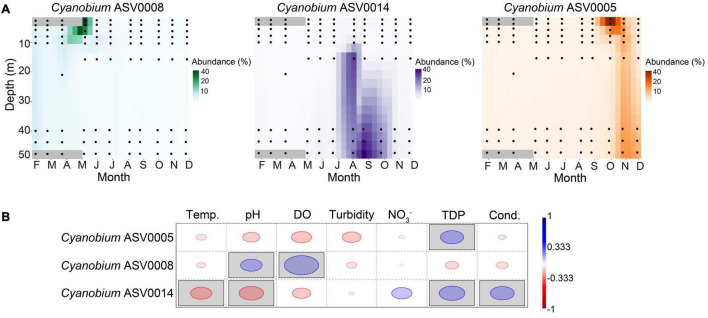
Dynamics of the three most abundant *Cyanobium* ASVs and correlation with environmental parameters. **(A)** Heatmaps show the spatiotemporal distribution across the water column of Lake Tiefer See. Data were interpolated for 10 days and 3 m water depth. Black circles represent the depths of water sample collection. **(B)** Rank-based Spearman correlation of the most abundant *Cyanobium* ASVs with lake physicochemical parameters in Lake Tiefer See. Gray-shaded squares are significant at *p* < 0.05 with Bonferroni *p*-value correction. Blue circles show positive while red circles show negative correlation. Temp. = Temperature, DO = dissolved oxygen, NO_3_^–^ = nitrate, TDP = total dissolved phosphorus, Cond. = conductivity.

We observed a potential niche separation among the three most abundant ASVs 0005, 0008, and 0014 assigned to *Cyanobium* (Figures 4B, 6A). Because of the different seasonal relative abundances among the *Cyanobium* ASVs 0005, 0008, and 0014, we used a rank-based Spearman’s correlation coefficient to check potential correlations of these ASVs with environmental parameters (Figures 6A,B). The ASV0005 which was dominant in fall had a significant positive correlation with TDP (*R*_*S*_ = 0.52), and negatively correlated to temperature, pH, turbidity and DO although insignificant (*R*_*S*_ > −0.4). ASV0008 which was most abundant in winter and spring correlated positively with pH (*R*_*S*_ = 0.48) and DO (*R*_*S*_ = 0.76). Lastly, the ASV0014 which dominated the hypolimnion in summer correlated positively with TDP and conductivity (*R*_*S*_ > 0.52) and negatively with temperature and pH (*R*_*S*_ > −0.47).

**FIGURE 7:**
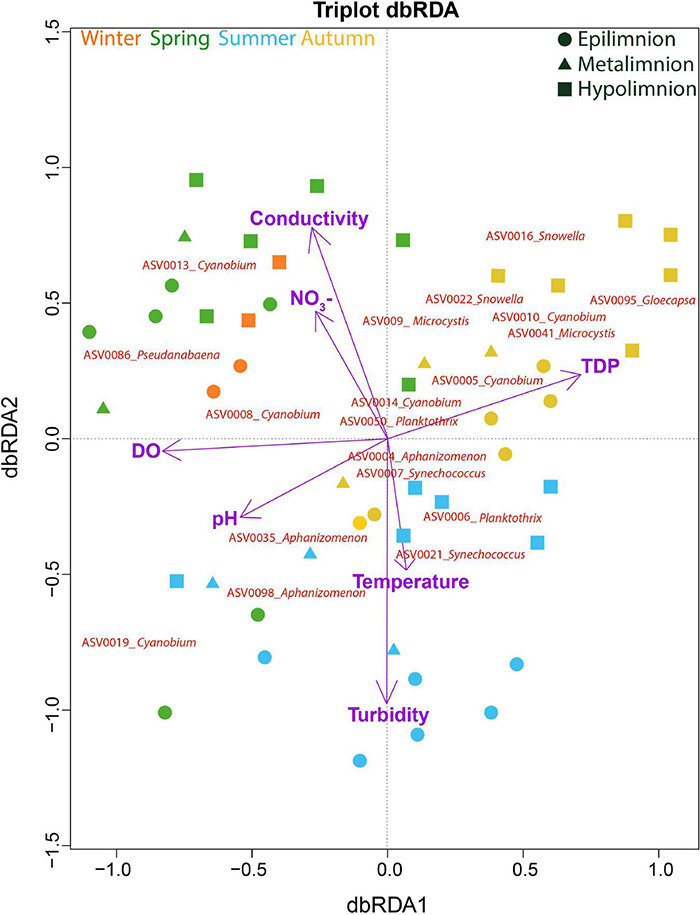
Cyanobacterial composition in relation to environmental variables. Distance-based Redundancy Analysis (dbRDA) on the effect of environmental variables on cyanobacteria community composition. The total variation explained by the dbRDA model was 48%. The samples are color-coded according to seasons. The environmental variables explaining the variation in cyanobacteria community were all significant (adjusted *R*^2^ = 0.34) and are projected as purple vectors. The response variables are shown at the ASV level.

**FIGURE 8:**
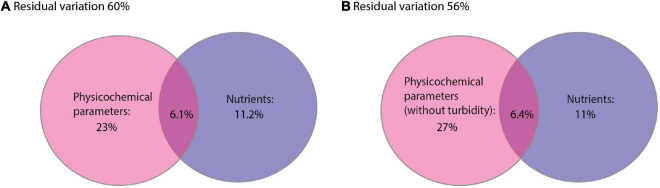
Venn diagrams displaying the results of the variation partitioning analysis (VPA). The two explanatory matrices used contained variables on lake internal physicochemical parameters **(A)** (temperature, dissolved oxygen, turbidity, conductivity and pH) and nutrients (total dissolved phosphorus and nitrate). **(B)** (temperature, dissolved oxygen, conductivity and pH) and nutrients (total dissolved phosphorus and nitrate). Each circle represents the portion of variation accounted for by each explanatory matrix or a combination of them. The significance of the fractions of variability explained by the categories was determined via a Monte Carlo permutation test (*n* = 999).

A dbRDA was used to ascertain the environmental parameters responsible for the variation in the general cyanobacterial seasonal communities (Figure 7). The dbRDA model explained 48% of the total observed community variation and identified temperature, turbidity, TDP, NO_3_^–^, pH, conductivity and DO as environmental variables that significantly shaped cyanobacterial communities in Lake Tiefer See over the investigated seasons (Table 1). Furthermore, canonical VPA (Figure 8) was used to determine the percentage of variation explained by nutrients, physicochemical parameters as well as their combined effect on cyanobacterial population. The physicochemical parameters (23%; temperature, DO, turbidity, conductivity, and pH) and nutrients (11.2%; TDP and NO_3_^–^) were the groups of factors that significantly explained most of the variability in cyanobacteria composition in Lake Tiefer See. The interaction between both groups accounted for 6.1% of community variation, while 60% of the variation remained unexplained. Turbidity was not included in the VPA model because it has been shown to be triggered by both abiotic sediment resuspension in the bottom waters and biotic biomass production in the upper waters. However, when turbidity is included as part of the physicochemical parameters, total amount of variation explained by the VPA model increases to 45% (accounting for an additional 5%).

**TABLE 1:** Summary of significant explanatory parameters used in the distance-based redundancy analysis (dbRDA) model.

**Explanatory variable**	**AIC**	**Pseudo-*F***	***p***
DO	159.73	8.77	0.001
Conductivity	156.75	4.92	0.002
Turbidity	157.26	4.92	0.002
TDP	154.76	4.56	0.002
pH	153.98	4.42	0.005
NO_3_^–^	152.98	3.56	0.004
Temperature	150.95	3.07	0.008

*The model explained 48% of the total variation (p = 0.001). The significance of the environmental variables was tested by 999 Monte Carlo permutations and were selected by forward selection (Adjusted R^2^ = 0.34). AIC, Akaike information criterion.*

## Discussion

Altogether the cyanobacteria community in Lake Tiefer See is dominated by the orders Synechococcales and Oscillatoriales indicative of its oligo-mesotrophic status (Callieri et al., 2012a; Soule and Garcia-Pichel, 2019). This aligns with observations in several natural and artificial lakes e.g., peri-Alpine lakes (Monchamp et al., 2018), sub-Alpine deep and shallow lakes as well as reservoirs and fish ponds (Vörös et al., 1998). However, our study shows a previously unknown cyanobacteria inter-species spatio-temporal variation in a temperate hard-water lake. Although this study spanned a monomictic lake circulation mode, its results can be extrapolated to years with similar circulation modes provided other lake environmental parameters remain the same. This is based on the premise that despite the potential annual variability in inter-species relative abundances which are mainly influenced by fluctuations in lake environmental parameters, a general pattern would still be observed. That is, cyanobacteria seasonal variation would typically begin with an increase in abundance of nitrogen-fixing taxa which utilize sediment resuspended nutrients made available during water turnover in spring (e.g., *Aphanizomenon*), then a breakdown in their population in summer e.g., by fungal parasitism (Gerphagnon et al., 2015), and followed by an increase in abundance of non-nitrogen fixers e.g., *Planktothrix* sp. at the base of the euphotic zone or in the epilimnion (Walsby and Schanz, 2002).

### Succession and Seasonal Variation of Cyanobacterial Communities

The observed seasonal variation of the CCC suggests the formation of distinct subpopulations in Lake Tiefer See dominated by a few taxa belonging to Synechococcales (*Cyanobium, Snowella*, and *Synechococcus*), Nostocales (*Aphanizomenon-* spring to summer), Chroococcales (*Microcystis-* fall), and Oscillatoriales (*Planktothrix-* late spring to fall). The interplay of abiotic factors and nutrients such as temperature, turbidity, DO, pH, conductivity, NO_3_^–^, and TDP were likely responsible for shifting the CCC and the abundance of the dominant taxa (Figures 7, 8 and Table 1). This observation adds to previous amplicon-based CCC studies in shallow and deep temperate lakes (Diao et al., 2017; Salmaso et al., 2018). Unlike in the nearby Lake Stechlin, where *Dolichospermum circinale* and *Aphanizomenon flosaquae* were the dominant cyanobacteria taxa (Dadheech et al., 2014), *Cyanobium*, *Synechococcus*, and *Planktothrix* were most abundant in Lake Tiefer See (Figures 3, 4B). The dominance of these taxa in Lake Tiefer See may be attributed to physiological advantages like rapid growth (*Cyanobium*, *Synechococcus*), colony formation (*Cyanobium*), and cell buoyancy regulation (*Planktothrix*, Nwosu et al., 2021b). Consequently, these taxa are able to better adapt to the seasonally changing in-lake conditions (Reynolds et al., 1987; Callieri and Stockner, 2000; Carey et al., 2012). Our observation of higher abundance of potential toxin-producing taxa (*Planktothrix, Aphanizomenon*, and *Microcystis*) accompanied by a lower picocyanobacterial abundance (*Cyanobium* and *Synechococcus*) is in line with that from other temperate lakes with varying depths and trophic status (Callieri et al., 2012b). *Planktothrix, Aphanizomenon*, and *Microcystis* were shown to cause surface blooms either directly because of anthropogenic nutrient loading e.g., via agriculture (Paerl and Huisman, 2008; Sukenik et al., 2015) or indirectly by continuous warming (Wagner and Adrian, 2009; Winder et al., 2009).

The seasonality of cyanobacteria observed in Lake Tiefer See is similar to known succession patterns in various temperate lakes which typically begins in spring when picocyanobacteria and nitrogen-fixing taxa (e.g., *Aphanizomenon* that survived the winter in sediments) become dominant by taking advantage of increasing temperature, light intensity, and the nutrients made available during spring mixing of the lake (Walsby, 2005; Hense and Beckmann, 2006; Kaiblinger et al., 2007). This leads to a cyanobacterial bloom evidenced by an increase in the total cyanobacteria abundance in the upper waters (epi- and metalimnion) in early spring likely associated with increase in bio-productivity, oxygen concentrations and the higher pH of the epilimnion (Figures 2B,D, 4B; Roeser et al., 2021). The onset of stratification in Lake Tiefer See as seen from the temperature, DO and other parameters in the upper water column (i.e., mid-April, Figure 2B), follows an increase in bio-productivity from the aforementioned cyanobacteria groups that grow optimally as temperature begins to increase. The breakdown of fall cyanobacteria populations (e.g., picocyanobacteria and *Aphanizomenon*) is likely due to factors such as viral lysis (Personnic et al., 2009), increased epilimnion turbidity resulting from biomass production in spring, subsequent lower light intensity, nutrient depletion, predation and grazing by zooplankton communities like rhizopods, ciliates, rotifers (Gerphagnon et al., 2015), *Daphnia* (Urrutia-Cordero et al., 2016), copepods (Ger et al., 2016), and/or parasitic fungi (Jia et al., 2010). In fall, the concurrent resuspension of shallow-water sediments together with the release of phosphorus into the water column favored a second cyanobacteria bloom (Figure 2H). Our data show that this bloom comprises mainly cyanobacterial ASVs assigned to *Microcystis, Snowella, Planktothrix*, and *Cyanobium*.

The peak in total cyanobacteria abundance in late August in the metalimnion (10 m; Figure 4A) may have resulted from the presence of a density gradient zone during lake stratification that prevent complete downward transport of the CCC from the water column to the sediments, especially of buoyant and filamentous taxa like *Planktothrix* (Halstvedt et al., 2007; Boehrer and Schultze, 2008). Also, the peak in summer cyanobacteria abundance in upper waters aligns with previous findings which identified temperature as an important factor leading to increase of cyanobacteria populations in lakes in central and northern European regions (Richardson et al., 2018). Considering that the copy numbers of the 16S rRNA gene among cyanobacteria genera have a low range in variability (2.7 ± 1.0 copies; Větrovský and Baldrian, 2013), we do not think the abundance and sequencing data from our study are skewed.

We further observed that seasonal changes significantly altered species richness especially in the epi- and metalimnion in Lake Tiefer See (Figure 4). The increase in water temperature, light and nutrient availability likely explains the significant increase in species richness between spring and summer in the epi- and the metalimnion. In addition, the Shannon species diversity minima in spring (epilimnion) and summer (meta- and hypolimnion) are attributed to the dominance of a few ASVs assigned to *Cyanobium* and *Planktothrix* and supported by the uneven species distribution during these seasons (Supplementary Figure 3). An environmental factor that likely affects the CCC in Lake Tiefer See is the lake circulation mode which depends on the formation of a winter ice cover. Thus, our results show that the lake mixing regime is an important environmental factor that affects the CCC in Lake Tiefer See. Further investigation of the CCC covering longer periods with potentially different lake mixing regimes would be necessary to compare and trace possible changes in cyanobacteria dynamics driven by water circulation mode.

### Picocyanobacterial Dynamics

The abundance of the picocyanobacterial *Cyanobium* and *Synechococcus* observed in Lake Tiefer See followed a bimodal pattern common in most temperate lakes, it means, a spring or early summer peak and a second peak in late summer or fall (Stockner et al., 2006). The bimodal picocyanobacterial abundance observed in Lake Tiefer See concurs with previous observations in the nearby oligo-mesotrophic Lake Stechlin (Padisák et al., 2003) and/or in large subalpine lakes such as Lake Constance (Gaedke and Weisse, 1998) and Lake Maggiore (Callieri and Piscia, 2002). The peak abundance of ASVs assigned to *Synechococcus* (in June) and *Cyanobium* (in October; see Figure 4B) at different water depths throughout the year may be explained by their ability to tolerate and adapt to a variety of light conditions (Gervais et al., 1997; Callieri and Piscia, 2002). Other abiotic factors driving picocyanobacterial variability include nutrient availability (Bell and Kalff, 2001), lake morphometry and thermal regime (i.e., deep lakes with a stable vertical structure promote picocyanobacterial development, Camacho et al., 2009), changes in lake physicochemical factors that promote the coexistence of phycoerythrin- and phycocyanin-rich picocyanobacterial groups (Postius and Ernst, 1999; Stomp et al., 2007), and the interplay between ultraviolet and photosynthetically active radiation (Callieri et al., 2001, 2012b). Additionally, variations in picocyanobacterial dynamics could be driven by biotic factors such as grazing by ciliates and protists (Callieri et al., 2002; Jezberová and Komárková, 2007) as well as viral lysis (Personnic et al., 2009). Based on the low Chl*a* values (< 20 μg L^–1^ in 2019; Figure 2E) from Lake Tiefer See and thus its oligo-mesotrophic status, we suggest that there is likely an enrichment of phycoerythrin picocyanobacteria population in Lake Tiefer See. This would be in line with a European multi-lake series investigation comprising deep and shallow lakes which revealed that phycoerythrin-rich picocyanobacteria dominated the water column at low Chl*a* levels (Vörös et al., 1998).

The seasonal and spatial (i.e., water depth) differences in the relative abundances of the *Cyanobium* ASVs 0005, 0008, and 0014 suggests niche specialization within this genus. The spring epilimnetic peak abundance (>40%) of *Cyanobium* ASV0008 suggests that it copes well at 10–15°C and it may also contribute substantially to the increase in pH values (up to 9). This is in line with monitoring reports from Lake Tiefer See that showed the increase in phytoplankton bio-productivity in spring increases the pH value of the water up to 8.6 (Roeser et al., 2021).

In Lake Tiefer See, the thermal stratification in summer seems to establish a niche for *Cyanobium* ASV0014 in the hypolimnion (∼15–50 m) because of a high relative abundance (40%) of this ASV, compared to the upper waters or other seasons where it was generally ≤ 15%. While it has been suggested that temperature plays a key role in initiating picocyanobacterial abundance and growth in freshwater ecosystems (Weisse, 1993; Vörös et al., 2009), its effect on regulating picocyanobacterial dynamics is still unclear (Callieri et al., 2012a). The negative correlation of ASV0014 with temperature suggests that this taxon copes better with lower temperatures in the hypolimnion than other groups. In addition, ASV0014 might utilize the most penetrating green and blue lights in the lower meta- to upper hypolimnion to outcompete other autotrophs and promote its success in the low-light reaching deeper waters in summer (Pick and Agbeti, 1991; Vörös et al., 1998; Callieri et al., 2007). Elsewhere, higher picocyanobacterial abundances have also been reported in the lower meta- to upper hypolimnion in Lake Stechlin (Padisák et al., 2003) and Lakes Huron and Michigan (Fahnenstiel and Carrick, 1992). In October, when thermal gradient diminishes caused by lower temperatures in the water column, ASV0005 became the most abundant *Cyanobium* ASV. Similar to ASV0014 it is also negatively correlated to temperature albeit insignificant. This means the *Cyanobium* ASVs 0014 and 0005 may be psychrotolerant (cold-adapted) in nature able to tolerate lower temperatures better than other cyanobacteria groups. Their generally lower relative abundances during periods of warmer water temperature (11–20°C) poses the assumption that they may be “cold-water-preference” species. Psychrophilic picocyanobacterial taxa have been reported in various aquatic environments ranging from the temperate East China sea (Choi et al., 2013) to polar seas (Tang et al., 1997) and most recently in freshwater Lake Biwa, Japan (Cai et al., 2021). While the psychrotolerant picocyanobacteria observed in other freshwater ecosystems were either from surface water samples (Xu et al., 2015) or in the deeper epilimnion (5 m, Lake Biwa Japan), the *Cyanobium* ASVs 0014 and 0005 from Lake Tiefer See were also abundant in the meta- and hypolimnion (15–55 m). In addition, a further factor that possibly contributed to the increase in abundance of the *Cyanobium* ASVs 0014 and 0005, is the increase in TDP availability beginning in summer in the hypolimnion with a peak in fall. Also, the sediment re-suspension in the shallower parts of the lake may facilitate the release of phosphorus (Nuhfer et al., 1993). Our data suggest that *Cyanobium* ASVs 0005 and 0014 may be the main utilizers of this nutrient. Since the cyanobacteria community data from our study are based on relative abundances, the observed hypolimnetic abundance of *Cyanobium* ASV0014 in summer could also result from a decrease of other ASVs. Nevertheless, even if the other ASVs were only reduced while ASV0014 was “maintained/stable,” it still indicates that it is able to cope well with the prevailing ambient conditions in deeper waters.

Altogether, the observed fine-scale spatiotemporal dynamics of the picocyanobacteria is a clear indication for niche separation in the water column of deep temperate hard-water lakes especially of individual taxa within *Synechococcus* and *Cyanobium*. Future studies could combine isolation, cultivation and physiological tests as well as metagenomics to get a more complete insight on the picocyanobacterial dynamics we observed here.

### *Planktothrix* Dynamics

The *Planktothrix* ASV0006 in Lake Tiefer See showed a peak abundance in the zone of low light intensity (0.1%) in the metalimnion which concurs with observations in a number of European lakes such as Lake Pusiano (Legnani et al., 2005), Lake Zürich (Posch et al., 2012), Lake Bourget (Jacquet et al., 2005) Lake Stechlin (Dadheech et al., 2014), and Lake Steinsfjorden (Halstvedt et al., 2007). The results from our molecular approach are also substantiated by similar findings based on microscopic (Posch et al., 2012; Selmeczy et al., 2016) and cell count studies (Jacquet et al., 2005; Wentzky et al., 2019). The late summer to fall peak in the relative abundance of *Planktothrix* in the metalimnion of Lake Tiefer See may be explained by factors such as low light intensity (0.1%) sufficient for photosynthesis (Walsby and Schanz, 2002; Walsby et al., 2006), lower temperatures (<10°C) and buoyancy adjustments in the face of changing light dynamics (Konopka, 1982; Teubner et al., 2003; Walsby et al., 2004). The presence of *Planktothrix* at all investigated water depths, even at 45 m depth both during and after thermal stratification, indicates an acclimation to water temperatures as low as 5°C, thus ensuring their winter survival (Walsby, 2005; Holland and Walsby, 2008; Dokulil and Teubner, 2012).

### Deep Chlorophyll Maxima and Metalimnetic Oxygen Minimum

In Lake Tiefer See, the deep chlorophyll maxima (DCM) formed by *Cyanobium* ASV0008/*Synechococcus* (7 m; spring), *Cyanobium* ASV0005 (7 m; fall), and *Planktothrix* (10–15 m; spring to fall) probably result from the adaptation of these taxa to both low nutrients and light intensity in the thermocline (Walsby et al., 2004; Callieri et al., 2007). This suggests ecotype and niche specialization as well as coexistence among the DCM-forming taxa in Lake Tiefer See. The coexistence may be explained by their different responses to environmental factors such as light and nutrient (NO_3_^–^ and TDP) availability (Selmeczy et al., 2016). For example, the requirements for different degrees of light intensity might explain the *Planktothrix* and *Synechococcus* summer peak relative abundances at 10 m and 7 m water depths, respectively. In the nearby Lake Stechlin spatial segregation and coexistence of DCM forming cyanobacteria was reported with *Aphanizomenon* as a main contributor (Selmeczy et al., 2016). Similar observations of the coexistence and niche specialization among cyanobacterial taxa with red and green pigments along the light gradient were reported for several aquatic ecosystems (Pick and Agbeti, 1991; Vörös et al., 1998; Stomp et al., 2007). The heterotrophic decomposition of the DCM-forming taxa as indicated by the qPCR results would explain consumption of dissolved oxygen (11 and 13 m; Figure 2B) between August and November (Gerphagnon et al., 2015). The development of a metalimnetic oxygen minimum indicates that enhanced particle retention time in this relatively denser region favors heterotrophic decomposition of organic matter (Wentzky et al., 2019; Mi et al., 2020).

## Conclusion

This study provides the first detailed seasonal survey of the dynamics of CCC, spatial distribution and abundance in the deep hard-water Lake Tiefer See. Combining high-throughput sequencing and ASV analysis enables to reveal freshwater cyanobacteria dynamics on a high taxonomic resolution and suggests niche separation and coexistence of individual cyanobacterial species. At the same time, the study underlines limitations of purely DNA-based pelagic surveys in providing evidence for potentially novel, cold-adapted cyanobacterial species whose occurrence is suggested here.

## Data Availability Statement

The datasets presented in this study can be found in online repositories. The names of the repository/repositories and accession number(s) can be found below: https://www.ebi.ac.uk/ena, PRJEB40406, ERS5083566–ERS5083644.

## Author Contributions

ECN, AB, ED, and SL: conceptualization. ECN, PR, SL, and AB: methodology. ECN and SY: software. ECN, SL, and AB: validation. ECN, PR, SP, SY, and LG: formal analysis. ECN, PR, and SP: investigation. ECN, SP, and SY: data curation. ECN and PR: visualization. ECN: manuscript writing with input from all authors. SL: supervision. AB, DW, and SL: project administration.

## Conflict of Interest

The authors declare that the research was conducted in the absence of any commercial or financial relationships that could be construed as a potential conflict of interest.

## Publisher’s Note

All claims expressed in this article are solely those of the authors and do not necessarily represent those of their affiliated organizations, or those of the publisher, the editors and the reviewers. Any product that may be evaluated in this article, or claim that may be made by its manufacturer, is not guaranteed or endorsed by the publisher.

## Footnotes

1
http://shiny.raccoome.de/bubblePlot/
