# Supplementary material for: Species-Level Spatio-Temporal Dynamics of Cyanobacteria in a Hard-Water Temperate Lake in the Southern Baltics
Source: Front Microbiol. 2021 Oct 29;12:761259. doi: 10.3389/fmicb.2021.761259 (PMC8586417; doi:10.3389/fmicb.2021.761259)
Supplement: Supplementary file 1 [file Data_Sheet_1.docx]

***Supplementary Material***

**Species-level spatio-temporal dynamics of Cyanobacteria in a hard-water temperate lake in the southern Baltics**

Ebuka Canisius Nwosu*, Patricia Roeser, Sizhong Yang, Lars Ganzert, Sylvia Pinkerneil, Achim Brauer, Elke Dittmann, Dirk Wagner and Susanne Liebner

*** Correspondence:** [enwosu@gfz-potsdam.de](mailto:enwosu@gfz-potsdam.de)

This file includes:

**Supplementary Figures**

**Figure S1**. Most abundant cyanobacteria taxa and sample-wise cyanobacteria taxonomic

distribution dynamics.

**Figure S2**. Non-metric multidimensional scaling (NMDS) showing the

spatial and temporal variability of cyanobacteria amplicon sequence variants (ASVs ≥1%)

from this study. Bra-Curtis distance was used. Stress = 0.11.

**Figure S3**. Cyanobacteria species evenness in Lake Tiefer See

**Supplementary Tables**

**Table S1.** Results of bioinformatical analyses of sequencing data for each

sample, alpha diversity and sample barcodes.

**Table S2.** Seasonal one-way PerMANOVA pairwise analysis of cyanobacterial communities

**Table S3.** A two-way ANOVA showing that cyanobacteria species richness was impacted by

seasonality and not by lake stratification (epi-, meta-, and hypolimnion) and/or an interaction

effect of both factors. A Tukey post-hoc test showing pairwise analysis of cyanobacteria

species richness

**Supplementary Figures**

(A)
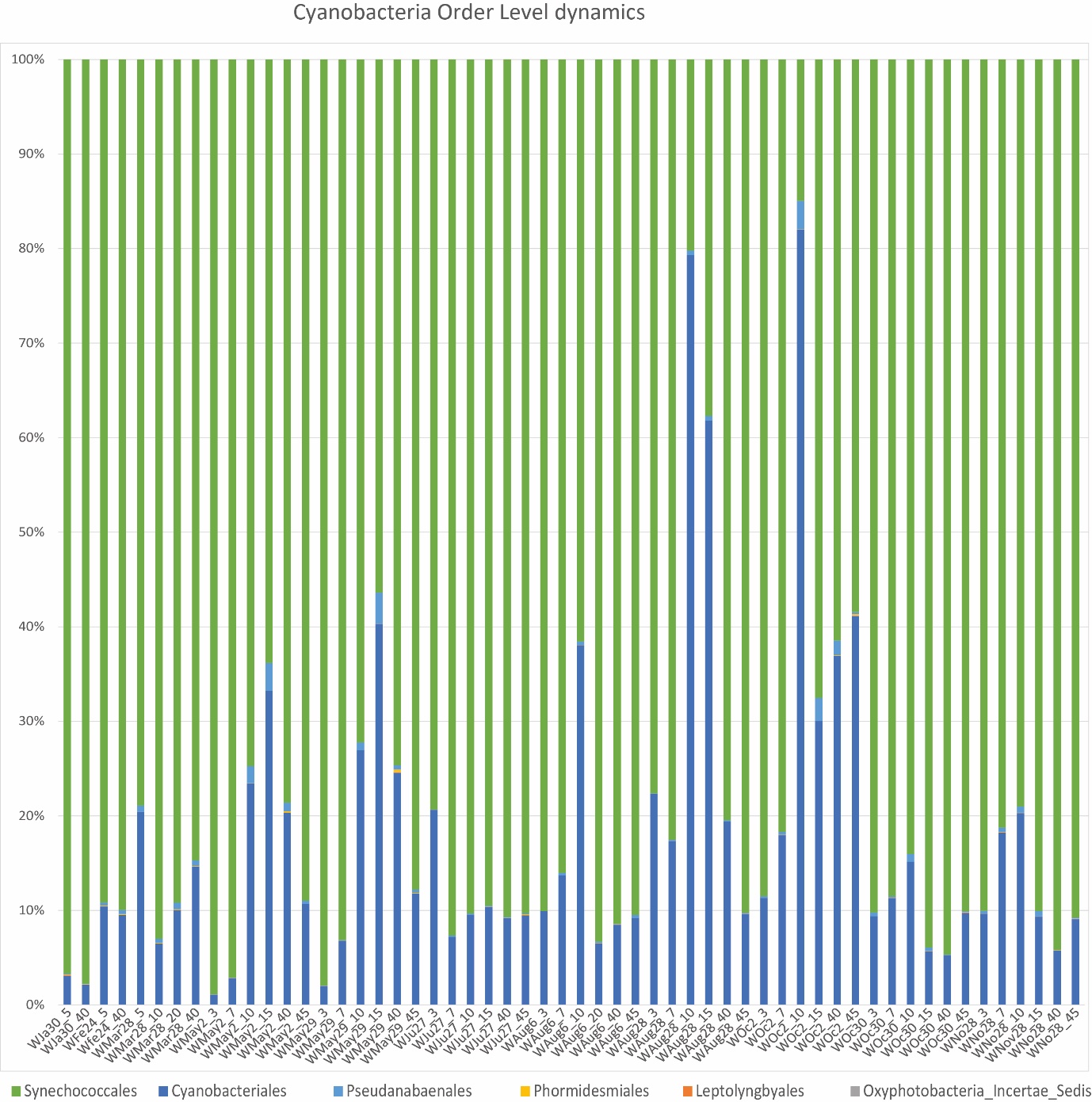


(B)
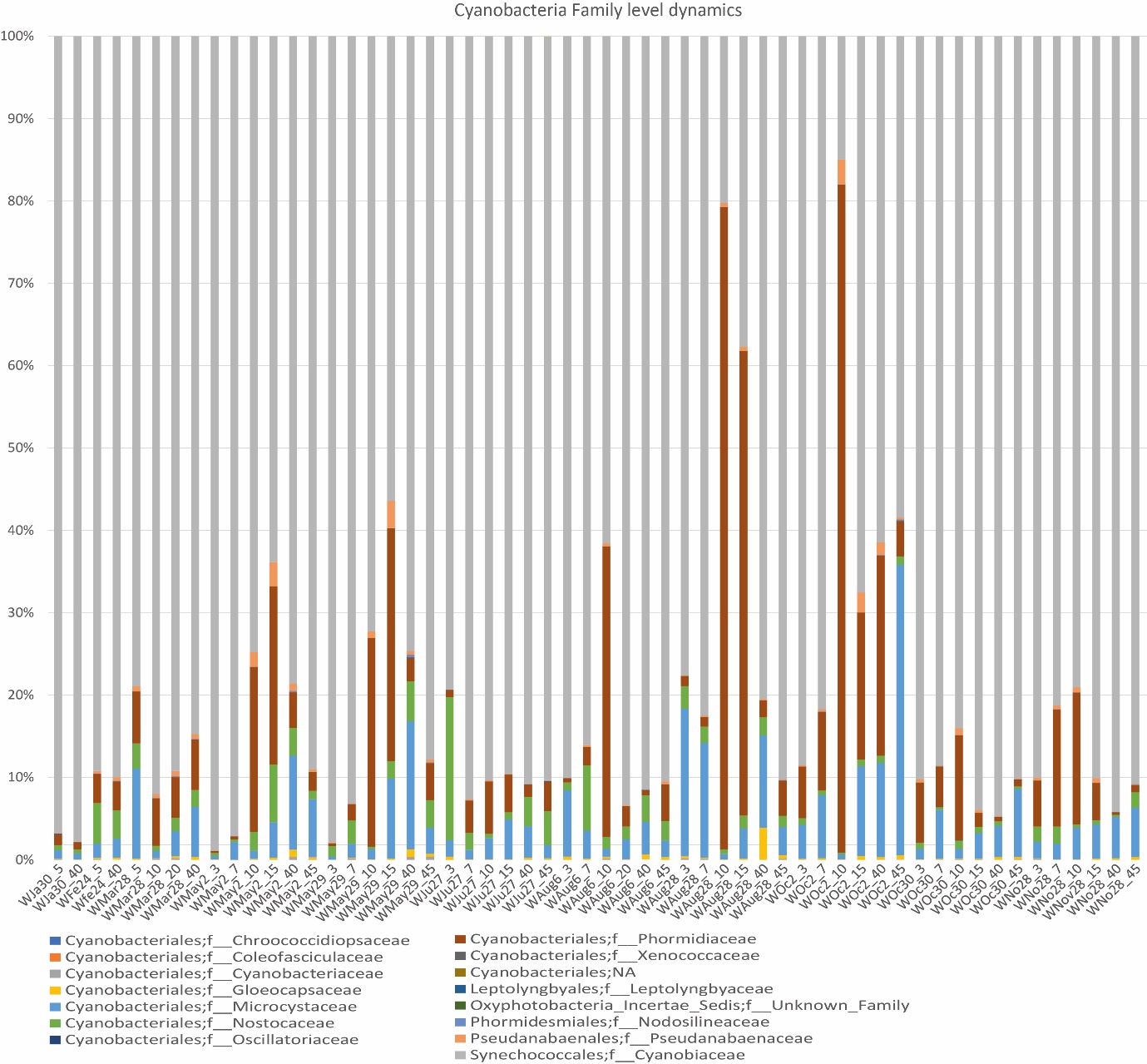


(C)
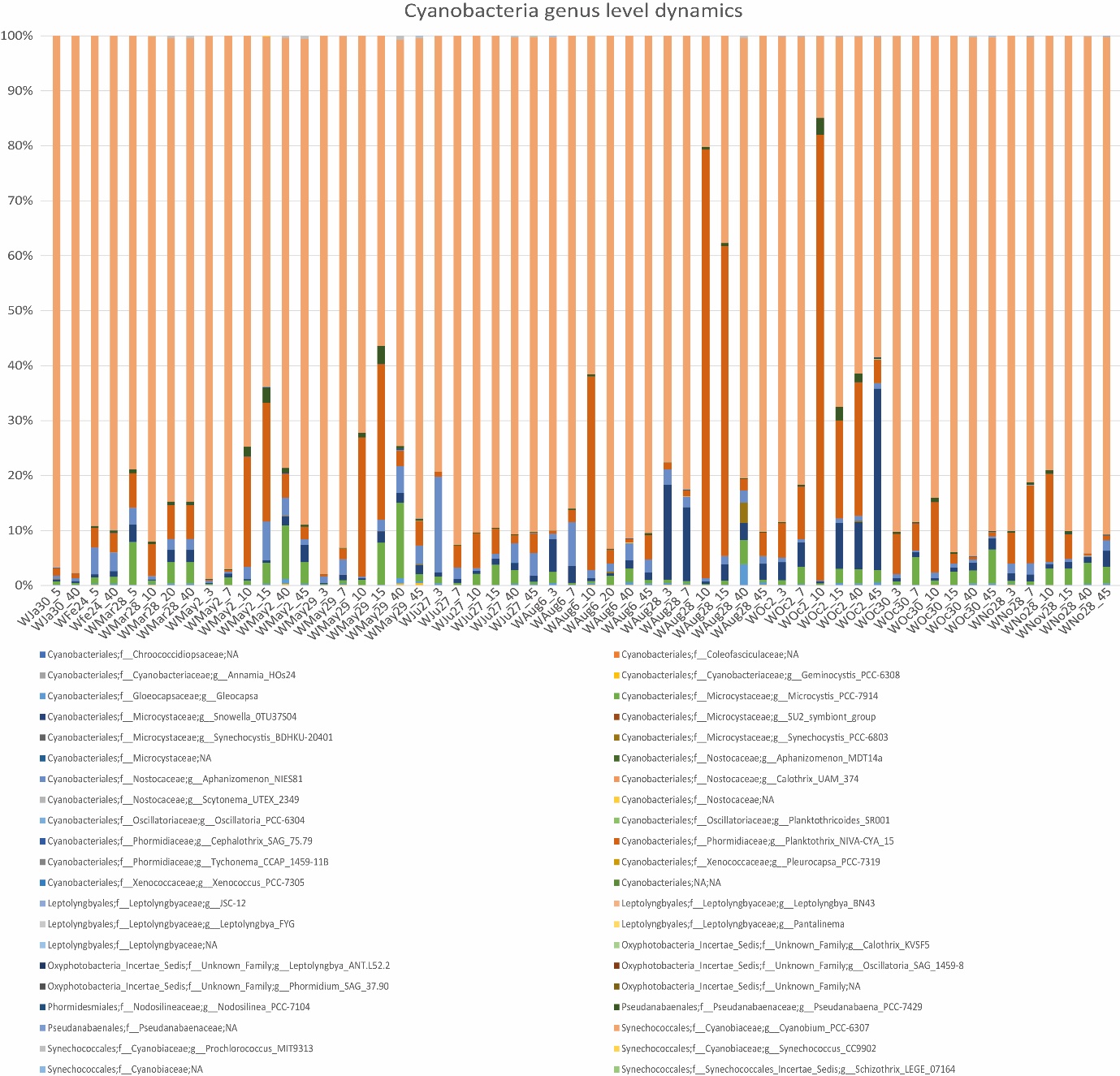


**Supplementary Figure S1**:

Barplots showing sample-wise cyanobacteria taxonomic distribution at the (A) Order level, (B)

Family level and (C) Genus level. Numbers beside months refer to sampling date while number

after the underscore refers to water depth, e.g., May2_10 refers to a sample collected at 10 m

water depth on 2^nd^ May.


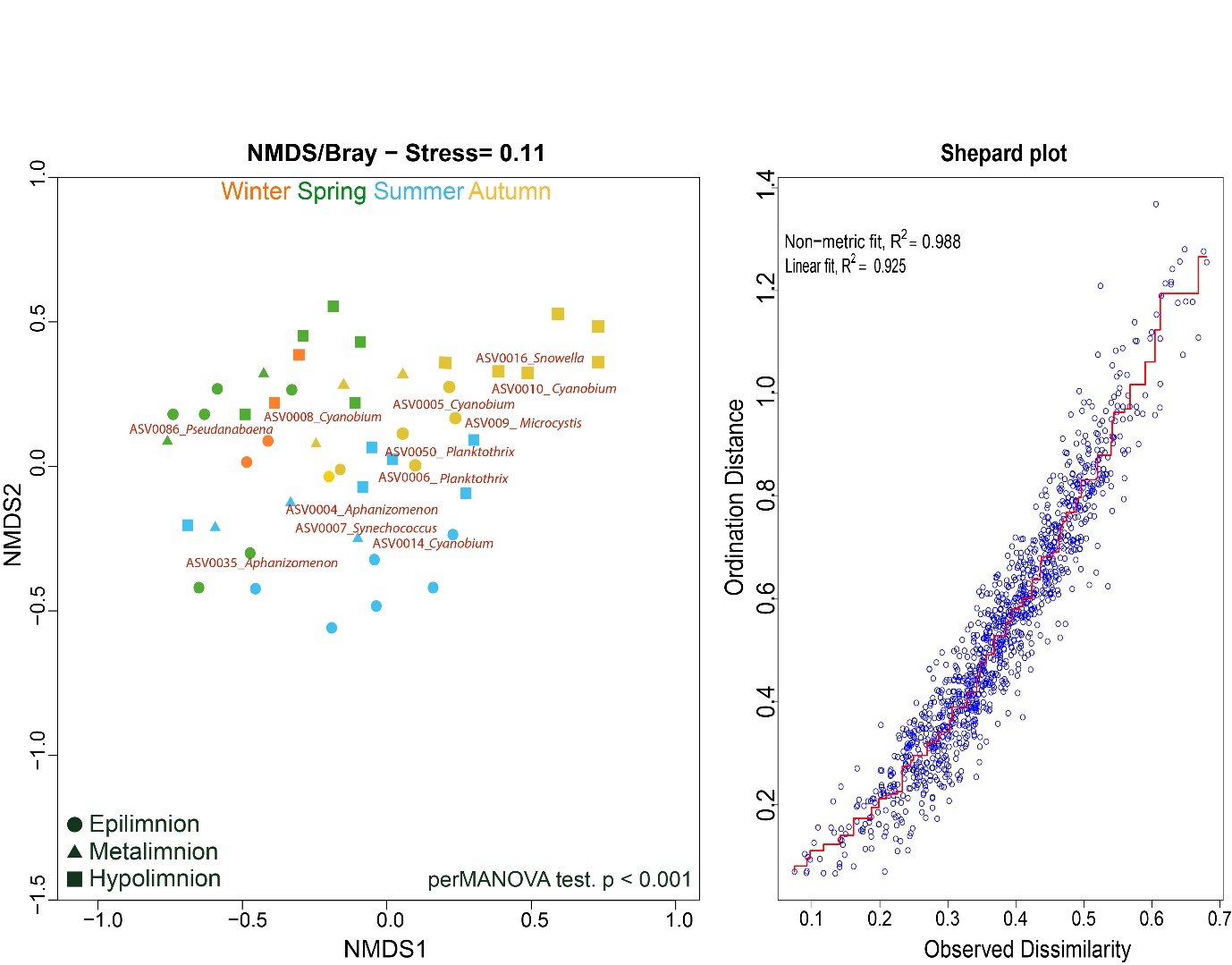


**Supplementary Figure S2**: Non-metric multidimensional scaling (NMDS) showing

spatiotemporal clustering patterns of cyanobacteria amplicon sequence variants (ASVs

≥0.1%) from this study. Bray-Curtis distance was used. Stress = 0.11. A nonparametric

PerMANOVA (Bray Curtis) was used to test the significance of the spatiotemporal clustering

Patterns. In the NMDS and PerMANOVA the sample seasons were predictors while

Hellinger-transformed cyanobacteria absolute read counts were the response variables.


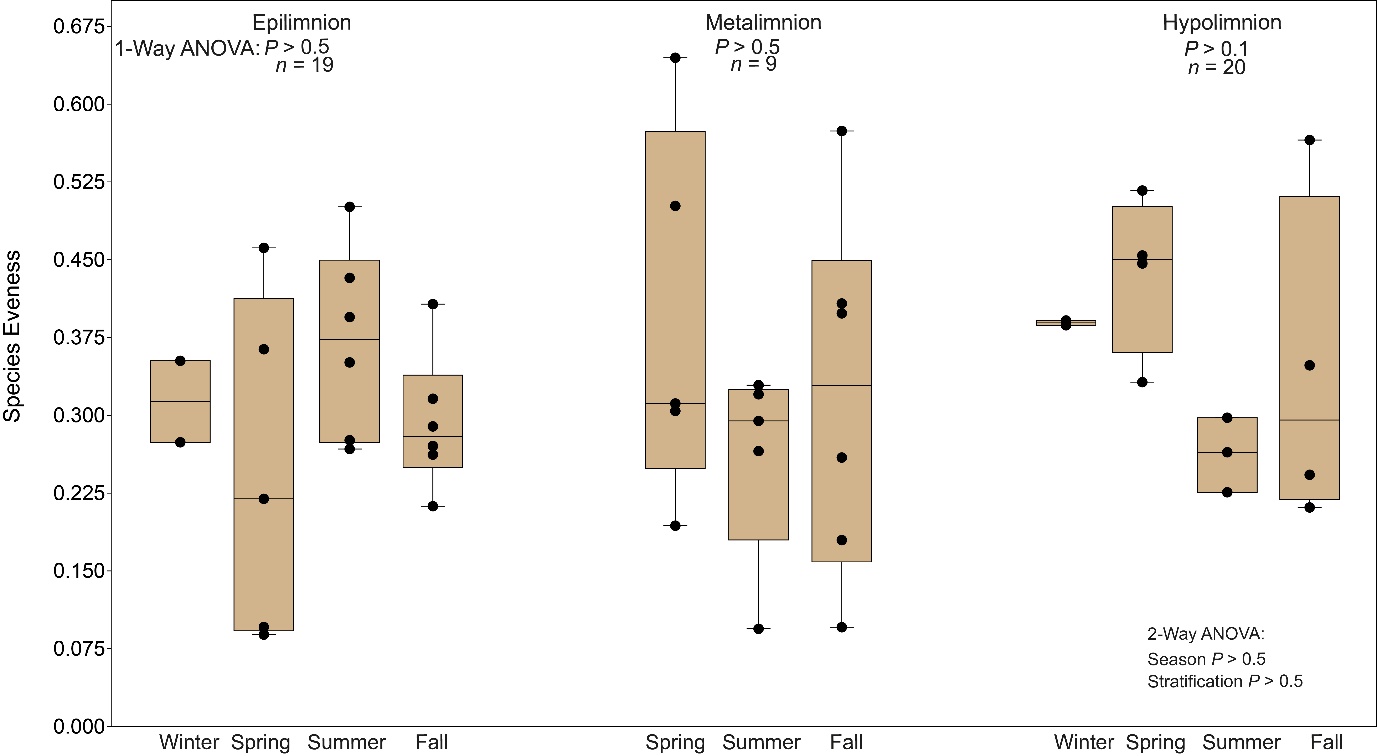


**Supplementary Figure S3**: Box plots showing the results of seasonal and stratification effects

on cyanobacteria species evenness in Lake Tiefer See via a two-way analysis of variance

(ANOVA) analysis. Test for significant seasonal variation in cyanobacterial species evenness

within the epi-, meta- and hypolimnion layers was tested via a one-way ANOVA.

**Supplementary Tables**

**Supplementary Table 1**. Results of bioinformatical analyses of sequencing data for each

sample, alpha diversity data for each sample and unique sample barcodes.

**Supplementary Table 2** One-way PerMANOVA on cyanobacterial communities from the

water column grouped into seasons. Summary presents overall test statistics. Pairwise analysis

shows Bonferroni corrected *P*-values above the diagonal and corresponding F-values below

the diagonal.

| **PERMANOVA** | **Statistics** | **Pairwise** | **Spring** | **Summer** | **Fall** | **Winter** |
| --- | --- | --- | --- | --- | --- | --- |
| **Permutation N:** | 9999 | Spring |  | 0.0001 | 0.0001 | 0.0166 |
| **Total sum of squares:** | 6.474 | Summer | 9.274 |  | 0.0001 | 0.0001 |
| **Within-group sum of squares:** | 4.531 | Fall | 18.61 | 10.46 |  | 0.0001 |
| **F:** | 10.72 | Winter | 2.815 | 6.178 | 12.75 |  |
| **p (same):** | 0.0001 |  |  |  |  |  |

**Supplementary Table 3**

A two-way ANOVA showing that cyanobacteria species richness was impacted by

seasonality and not by lake stratification (epi-, meta-, and hypolimnion) and/or an interaction

effect of both factors. A Tukey post-hoc test showing significant pairwise differences in

cyanobacteria species richness in between seasons and between the epi- and hypolimnion

layers. Significant *P*-values are in bold.

| Summary statistics |
| --- |
| Df Sum Sq Mean Sq F-value Pr(>F) |
| Season 3 501.0 167.01 8.046 **0.000238 ***** |
| Zone 2 133.0 66.49 3.203 0.050727 . |
| Residuals 42 871.8 20.76 |

TukeyHSD (Tukey multiple comparisons of means 95% family-wise confidence level)

Pairwise comparison of the seasons and stratification (epi-, meta-, and hypolimnion) predictors used in the ANOVA model. Underlined *p*-values show significant interactions.

| Season |
| --- |
| diff lwr upr p adj |
| Spring-Autumn -8.009524 -12.538392 -3.480656 0.0001449 |
| Summer-Autumn -2.200000 -6.650099 2.250099 0.5541521 |
| Winter-Autumn -4.866667 -11.724730 1.991397 0.2442977 |
| Summer-Spring 5.809524 1.280656 10.338392 0.0071634 |
| Winter-Spring 3.142857 -3.766578 10.052293 0.6198716 |
| Winter-Summer -2.666667 -9.524730 4.191397 0.7270511 |

| Zone |
| --- |
| diff lwr upr p adj |
| Hypo-Epi -3.688947 -7.234956 -0.1429389 0.0398735 |
| Meta-Epi -2.051963 -6.530955 2.4270282 0.5115038 |
| Meta-Hypo 1.636984 -2.805870 6.0798378 0.6463238 |
